# Midline crossing is not required for subsequent pathfinding decisions in commissural neurons

**DOI:** 10.1186/1749-8104-7-18

**Published:** 2012-06-06

**Authors:** Jennifer Bonner, Michael Letko, Oliver Brant Nikolaus, Lisa Krug, Alexandria Cooper, Benjamin Chadwick, Phoebe Conklin, Amy Lim, Chi-Bin Chien, Richard I Dorsky

**Affiliations:** 1 Biology Department, Skidmore College, Saratoga Springs, NY, 12866, USA; 2 Department of Neurobiology and Anatomy, University of Utah School of Medicine, Salt Lake City, UT, 84132, USA

**Keywords:** Robo, DCC, Zebrafish, Midline crossing, Axon guidance

## Abstract

**Background:**

Growth cone navigation across the vertebrate midline is critical in the establishment of nervous system connectivity. While midline crossing is achieved through coordinated signaling of attractive and repulsive cues, this has never been demonstrated at the single cell level. Further, though growth cone responsiveness to guidance cues changes after crossing the midline, it is unclear whether midline crossing itself is required for subsequent guidance decisions *in vivo*. In the zebrafish, spinal commissures are initially formed by a pioneer neuron called CoPA (Commissural Primary Ascending). Unlike in other vertebrate models, CoPA navigates the midline alone, allowing for single-cell analysis of axon guidance mechanisms.

**Results:**

We provide evidence that CoPA expresses the known axon guidance receptors *dcc*, *robo3* and *robo2.* Using loss of function mutants and gene knockdown, we show that the functions of these genes are evolutionarily conserved in teleosts and that they are used consecutively by CoPA neurons. We also reveal novel roles for *robo2* and *robo3* in maintaining commissure structure. When midline crossing is prevented in *robo3* mutants and *dcc* gene knockdown, ipsilaterally projecting neurons respond to postcrossing guidance cues. Furthermore, DCC inhibits Robo2 function before midline crossing to allow a midline approach and crossing.

**Conclusions:**

Our results demonstrate that midline crossing is not required for subsequent guidance decisions by pioneer axons and that this is due, in part, to DCC inhibition of Robo2 function prior to midline crossing.

## Background

Commissural axonal pathfinding across the ventral midline of the spinal cord relies on sequential interpretation of guidance cues, which change depending on whether the growth cone is ipsilateral to its cell body (early in the pathway) or contralateral (later in the pathway). Initially, commissural growth cones are attracted to the midline through Netrin and Sonic hedgehog signaling [1,2], while simultaneously being insensitive to repulsive signaling through Slit. Midline crossing is achieved through silencing of Netrin attraction through Robo activation [3,4] and increased sensitivity to Semaphorin and Slit [5]. As Slit signals are present on both sides of the ventral spinal cord, [6-9] responsiveness to Slit-mediated repulsion in the midline is tightly regulated to allow growth cones to cross. Rig-1/Robo3 has been demonstrated to allow growth cone entry into the midline via inhibition of Slit responsiveness [10]. Post-crossing Slit responsiveness is mediated by the classical Slit receptors Robo1 and Robo2. Robo1 mediates the initial exit of growth cones from the midline, and both Robo1 and Robo2 act to position axons after crossing [11,12]. Multiple splice isoforms of *Robo3* have been identified in zebrafish, mouse, and human, and produce proteins with different functions, including Robo3var1/var2, which are slightly different in their mature N-termini, and Robo3.1/3.2, which have slightly different C-termini [13-18]. In mouse, Robo3.1 inhibits Robo1/2 function to allow growth cones to enter the midline, while Robo3.2 is repelled by Slit and thereby responsible for post-crossing axonal positioning [18].

Reduction of Robo3 function can result in appropriate targeting in the absence of crossing and intact function in both mice and humans. This is particularly evident in precerebellar neurons [18-20]. However, ipsilateral pathfinding of these neurons was not analyzed in detail, nor was analysis performed at the single-cell level. One possible interpretation of these data is stochastic appropriate targeting of a pioneer neuron followed by selective fasciculation of follower axons, and retention of synapses through activity dependent mechanisms. Our study directly addresses whether pioneer neurons are responsive to guidance cues in the absence of midline crossing, and carefully characterizes the pathfinding of pioneer neurons in various Robo/DCC mutant conditions. In the zebrafish spinal cord, an average of one commissural neuron per hemisegment grows across the midline at early stages of development. Termed CoPA, this neuron serves as the pioneer commissural neuron in the spinal cord [21,22], unlike mammalian commissural spinal neurons, which extend axons as a population. Thus, as a pioneer that is temporally separated from its followers, CoPA is an excellent single-cell model of commissural pathfinding.

This study demonstrates the coordinated activities of DCC (Deleted in Colorectal Cancer) and Robo family members in CoPA pathfinding. In mammalian systems, commissural neurons become responsive to Slits and Semaphorins only after crossing the midline [5,10,11]. However, using an *in vivo* single-cell approach, we have determined that ventral growth and midline crossing are not required for subsequent pathfinding decisions that are mediated by Slit/Robo signaling and other factors. In mutant and gene knockdown conditions in which CoPA does not cross the midline, the axons behave as if midline crossing has occurred by extending in the appropriate directions. At least one mechanism that accounts for this finding is DCC inhibition of Robo2 in pre-crossing growth cones in wild-type embryos. We show that in the absence of *dcc* function, ipsilaterally projecting CoPA neurons are repelled by the midline in a *robo2-*dependent manner, behavior typically reserved for post-crossing neurons. Further, we have clear evidence that both Robo2 and Robo3 ensure that commissures occur in precise, repeated units along the length of the spinal cord, as in both Robo mutants CoPA pioneer axons ascend within the midline before crossing, creating commissures widened along the anterior-posterior axis.

## Results

### Expression of Robo and DCC guidance receptors during zebrafish primary spinal neurogenesis

**Figure 1:**
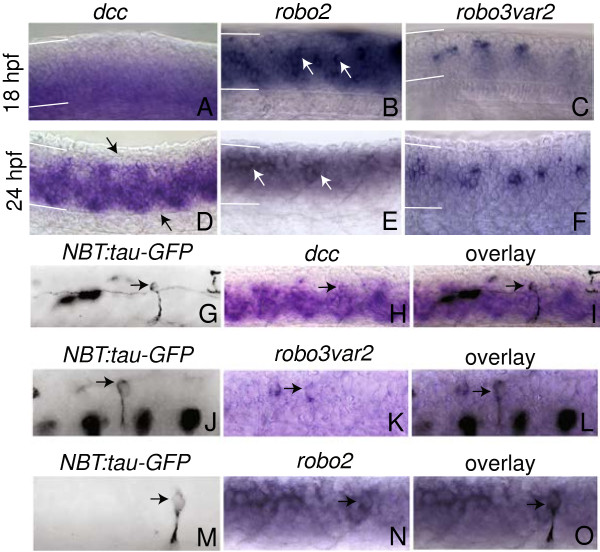
**Expression of *dcc*, *robo2 *and *robo3var2 *in the zebrafish spinal cord. (A**-**C)** Expression at 18 hpf. **A***dcc* is expressed in the ventral half of the spinal cord. **B***robo2* is expressed throughout the spinal cord and is observed in numerous postmitotic neurons (*arrows*). **C***robo3var2* expression is restricted to postmitotic neurons at 18 hpf. **D**-**F** Expression at 24 hpf. **D***dcc* expression has expanded to include almost the entire spinal cord; however, expression is weaker in segmentally repeated regions in dorsal and ventral spinal cord, as indicated by *arrows*. **E***robo2* is expressed in postmitotic neurons (*arrows*). **F** At 24 hpf, *robo3var2* is expressed in postmitotic neurons. **G**-**O** Pseudocolored reflected light image of anti-GFP immunofluorescence in *NBT:tau-GFP* embryos (**G**, **J**, **M**) and transmitted light images of the same embryos that have undergone *in situ* hybridization for *dcc, robo3var2,* and *robo2* (**h**, **k**, **n**) at 24 hpf. (**i**, **l**, **o**) exhibit overlain images of (**G** and **H**), (**J** and **K**), and (**M** and **N**), respectively. CoPA neurons are indicated by *arrows*. In all images, dorsal is *up*, anterior to the *left*. *White lines* indicate the dorsal and ventral boundaries of the spinal cord.

To determine if pioneer commissural neurons utilize guidance systems sequentially and cell autonomously, we characterized expression of known guidance receptors in the zebrafish spinal cord. The expression of three candidate axon guidance genes (*dccrobo2*, and *robo3var2*) was investigated because of known activities in commissural pathfinding in other animal models. DCC, Robo2, and Robo3 act distinctly to guide commissural neurons, though their combinatorial roles in a single commissural pioneer have not been determined. At the 18 somite stage (18 hpf), shortly after primary axons are first extended [22], *dcc* mRNA is diffuse and widespread throughout the ventral neural tube (Figure 1A). At the same stage, *robo2* is expressed throughout the neural tube (Figure 1B). Several classes of postmitotic neurons express *robo2* at this stage, suggesting that Robo2 may be actively guiding the axons of these early born neurons (Figure 1B). We also examined the *robo3var2* splice isoform because of its specific expression in postmitotic neurons during spinal cord development [14,16]. Consistent with previous reports, *robo3var2* is expressed in postmitotic neurons at 18 somites (Figure 1C; [14,16]).

By 24 hpf, many primary axons have reached their targets [22]. At this developmental stage, *dccrobo2*, and *robo3var2* continue to be expressed in the spinal cord. *dcc* mRNA expression is qualitatively increased in both postmitotic neurons and throughout the neural tube compared to 18 somites (Figure 1D). At 24 hpf, *robo2* is also expressed throughout the spinal cord, in postmitotic neurons (Figure 1E, [15]). *robo3var2* is found in segmentally repeated clusters of postmitotic neurons at 24 hpf, as previously reported (Figure 1F) [14,16]. The continuous expression of *dccrobo2*, and *robo3var2* implicates these genes in the pathfinding of neurons that are present between 18–24 hpf.

### Expression of Robo and DCC guidance receptors in CoPA pioneer neurons

In order to determine whether *dccrobo2*, and *robo3var2* are expressed in commissural pioneer (CoPA) neurons, simultaneous labeling of mRNA and neuronal morphology was performed. In 24 hpf embryos, CoPA was visualized using either stable *Tg(NBT:MAPT-GFP)*^*zc1*^ embryos or transient expression of the *NBT:Tau-GFP* plasmid used to make this transgenic line [23]. This transgene expresses axon-targeted GFP under control of the pan-neuronal *Xenopus Neuronal beta-tubulin* promoter. Anti-GFP immunofluorescence was performed simultaneously with mRNA in situ hybridization of *dccrobo2*, or *robo3var2*, and neuron identities were determined based on cell body location and morphology, and axon trajectories [21,24-27]. Consistent with our hypothesis that Robo and DCC family members play a role in CoPA guidance, we found that *dcc, robo3var2,* and *robo2* are all expressed in CoPA (Figure 1G-O). For each gene, expression was observed in >15 CoPA neurons.

### Robo2 is required to position CoPA axons in the dorsal-ventral axis after crossing the midline

**Figure 2:**
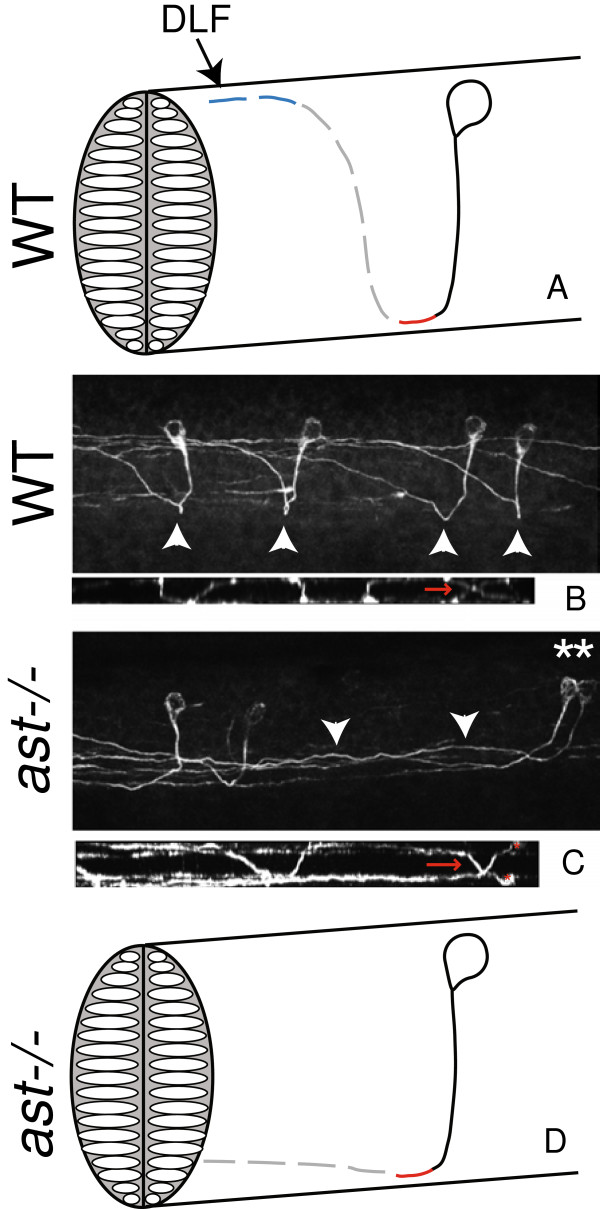
***robo2 *is required for escaping the midline and dorsal growth after crossing the midline. A** Schematic representation of one CoPA neuron. *Solid black line* indicates the ipsilateral ventral projection, *red line* indicates the midline crossing of the commissure, the *dotted gray line* indicates dorsal pathfinding after crossing the midline, and *dotted blue line* indicates anterior pathfinding after crossing the midline. CoPA axons join the dorsal longitudinal fasciculus (*DLF*) to ascend. CoPA axons extend ventrally at 17 hpf (*solid line*), cross the midline at 18 hpf, extend dorsally at 19 hpf, and grow toward the head at 21 hpf. Timeline adapted from Kuwada et al., 1990. **B** Confocal micrograph of 3A10 immunofluorescence in the spinal cord illustrating wild-type CoPA pathfinding in multiple segments, lateral view. *Arrowheads* indicate midline crossing or commissures. In smaller image below, a dorsal view of the same spinal cord indicates midline crossing (*red arrows*) **C** In *ast*^*ti272z*^ embryos, CoPA axons cross the midline, but remain ventral for several segments while ascending. *Asterisks* indicate affected CoPA cell bodies, and *arrowheads* mark axons that fail to extend dorsally after crossing the midline. In the smaller image below, a dorsal view of the same spinal cord indicates midline crossing (*red arrows*) of affected CoPA axons (cell bodies indicated by *asterisks*). **D** Summary diagram of *ast*^*ti272z*^ phenotype. In all images dorsal is *up*, anterior to the *left.*

During early stages of axon pathfinding in the zebrafish spinal cord, relatively few postmitotic neurons are present. CoPA pathfinding comprises four successive axon guidance decisions: ventral extension, midline crossing, dorsal growth away from the midline, and anterior growth. First, CoPA pathfinding in the spinal cord is initiated at 17 hpf, at which point it extends an axon ventrally. Second, CoPA crosses the midline in the ventral spinal cord at 18 hpf. Finally, after crossing the midline, CoPA extends in the dorsal-anterior direction at an oblique angle (at 19 hpf), and ascends to the dorsal spinal cord where it joins CoPA axons from other segments in the dorsal longitudinal fasciculus (DLF), at 21 hpf (Figure 2A-B, [22]). Other ascending commissural neurons arise later in development [21,22]. Thus, CoPA is the pioneer neuron that establishes the commissures in the spinal cord [21].

In order to determine if axon guidance mechanisms described in mammalian systems are conserved in zebrafish, CoPA pathfinding was analyzed in *robo2* (*ast*^ti272−/−^) homozygous mutants. To visualize CoPA, we performed immunofluorescence analyses with the 3A10 antibody, which has been used in zebrafish to label Mauthner neurons and labels spinal commissural neurons in mouse [28-32]. Our analysis focused on the 33 hpf developmental stage, at which CoPA pathfinding is complete, and 3A10 staining is strong and specific. While multiple commissural neuron subtypes are present in the spinal cord by this stage [21,22,26,27], the commissural neurons labeled by 3A10 were determined to be CoPA based on several criteria. These neurons uniquely exhibited anterior and posterior projecting dendrites, they lacked axonal branches in the ventral spinal cord, were present at a frequency of 0–2 cell bodies per hemisegment, and had a rostral-dorsal projection of post-crossing axons that occurs over 1–2 segments before joining the DLF (Figure 2B). All of these criteria are consistent with previous reports of CoPA characteristics [21,22,26]. To maintain consistency in analysis of CoPA pathfinding, we limited our examination to somite levels 9–12, analyzing up to 16 CoPAs per embryo. An embryo was scored for defective CoPA pathfinding if errors were observed in one or more CoPA neurons. Ns are given as numbers of scored embryos. In wild-type embryos, CoPA pathfinding defects were not observed.

The *ast*^ti272−/−^*robo2* allele contains a nonsense mutation in the extracellular domain [33]. While the zebrafish *robo2* mutant phenotype has been extensively studied by a number of investigators and Robo2 is well established as a guidance cue in zebrafish [30,32-37], the role of *robo2* in CoPA pathfinding has not been described. We found that CoPA neurons in *ast*^ti272−/−^homozygous mutants undergo normal ventral extension and enter the midline like their wild-type counterparts. However, once axons leave the midline and enter the contralateral spinal cord, they fail to grow dorsally in 98 ± 1.9% (*n* = 58) of mutant embryos (Table 1, Figure 2C). This is consistent with the role of Robo2 as a receptor for Slit that positions post-crossing axons [11] and establishes CoPA as a model for spinal commissural pathfinding at the single cell level. Since we were able to analyze the entire axon trajectory, we observed that affected *ast*^ti272−/−^ axons are able to recover and extend to the DLF in more anterior sections of the spinal cord (not shown). This suggests the presence of a dorsal attractive cue or additional midline repellent activity that is intact in these developmentally older sections of the spinal cord.

**Table 1:** CoPA pathfinding behaviors in various mutant/morphant conditions

**Strain**	**% Defective pathfinding, ±SEM**^**a**^	***N* =**
*robo3* mutant (*twt*^*tx209*^) heterozygous incross	54 ± 5.8	116
*robo3* mutant (*twt*^*tx209*^) heterozygous outcross	27 ± 9.2	65
*robo2* mutant (*ast*^*ti272z*^) homozygous incross	98 ± 1.9	58
*dcc* MO^c^	82.7 ± 4.6	157
*robo2* MO^b^	62.8 ± 2.9	177
*robo2* MO^c^	1.8 ± 1	177
*dcc* MO and *robo2* MO^c^	69.3 ± 5.2	169

^a^For details of CoPA defects, see text.

^b^Post-midline crossing defects.

^c^Pre-midline crossing defects.

### Midline crossing is not required for subsequent CoPA axon pathfinding

**Figure 3:**
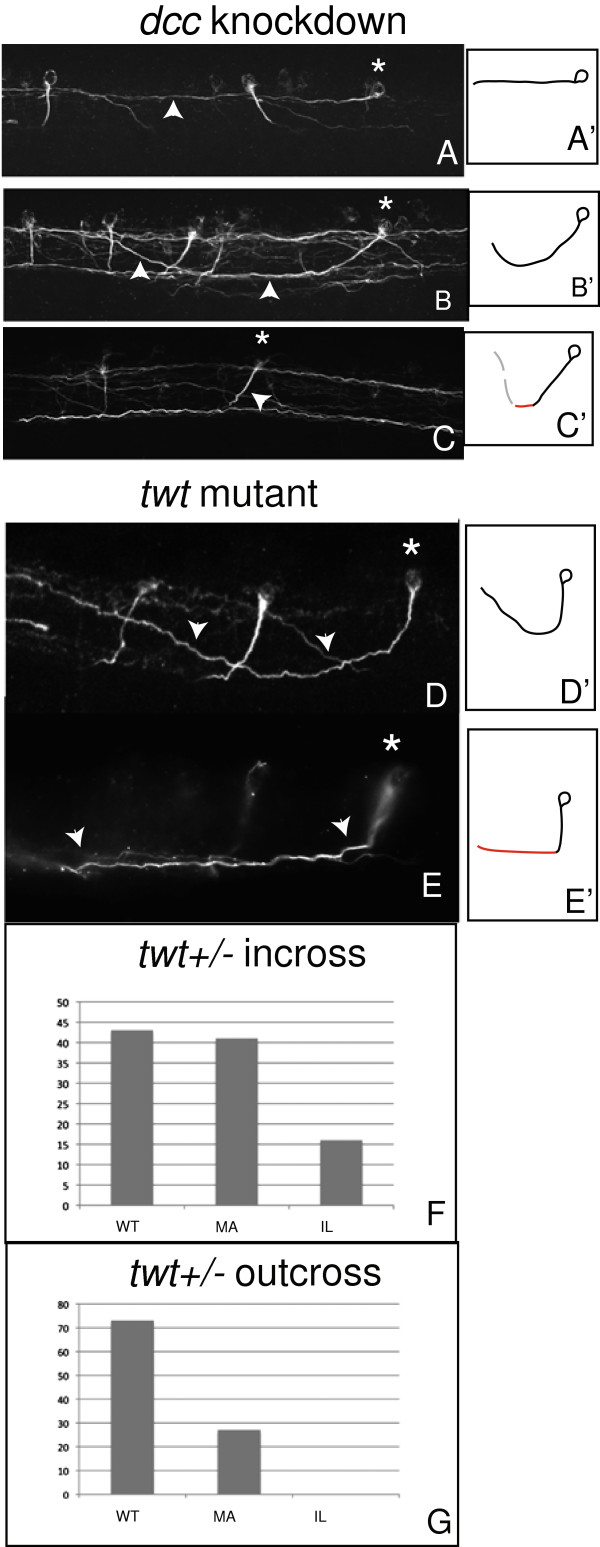
**Crossing is not required for reception of anterior or dorsal guidance cues. A**-**C***dcc* knockdown embryos demonstrating phenotypes consistent with loss of reception of attractive ventral cues, including anterior growth with a failure to grow ventrally (a’-c’) Summary diagrams illustrating affected axons. **A***Asterisk* indicates affected CoPA, while *arrowhead* points to affected CoPA axon. In (**B**), an ipsilateral projection that grows dorsally and anteriorly without crossing. *Asterisk* and *arrowheads* indicate affected cell body and axons, respectively. **C** A ventral extension (*arrowhead*) from an affected CoPA (*asterisk*) grows at an oblique angle to the ventral spinal cord (compare to wild-type pathfinding, Figure 2a-b). (**D**-**E**) *robo3* (*twt*^*tx209*^) embryos exhibit two distinct phenotypes. **D’**-**E’** Summary diagrams illustrating affected axons. In (**D**), *robo3* (*twt*^*tx209*^) CoPA axons fail to enter the midline and ascend instead on the ipsilateral side of the spinal cord. *Asterisk* indicates affected CoPA neuron, *arrowheads* indicate ipsilateral projection in this image, in which only the ipsilateral spinal cord was imaged. In (**E**) *robo3* (*twt*^*tx209*^), CoPA axons enter the midline but remain for some distance while ascending before exiting the midline on the contralateral side (“midline ascending”). *Asterisk* denotes CoPA cell body, *arrowheads* indicate axons ascending within the midline. In all images, dorsal is up, anterior to the *left*. (**F**-**G**) Comparative phenotypes from offspring of heterozygous incross (**F**) versus outcross to wild type (**G**). Only in offspring of heterozygous incross is the ipsilateral (*IL*) CoPA phenotype observed. The midline ascending (*MA*) phenotype is present in both conditions.

Based on evidence *in vitro* that midline crossing results in increased responsiveness to Slits and Semaphorins [5], we investigated whether midline crossing was a requirement for subsequent pioneer neuronal pathfinding *in vivo*. We hypothesized that the Netrin receptor DCC is required for midline crossing in zebrafish. To test this, a *dcc* translation blocking antisense morpholino oligonucleotide (MO) [38] was injected at the one cell stage. In *dcc* knockdown embryos, CoPA pathfinding was analyzed at 33 hpf using 3A10 immunofluorescence. We found that 82.7% (±4.6, *n* = 157, Table 1) of *dcc* morphants displayed one of three classes of CoPA pathfinding defects. In the first class, CoPA neurons failed to extend an axon ventrally, but extended an ipsilateral projection in the anterior direction (Figure 3A). In the second class of *dcc* morphant defects, CoPA axons extended ventrally, but failed to cross the midline. In spite of this, axons extended anteriorly and dorsally. We next tested whether the anterior growth in affected *dcc* morphants was stochastic or reflected active guidance mechanisms. As 84% (*n* = 19) of affected CoPA axons grew anteriorly, this does not support a stochastic mechanism and indicates that CoPA axons can respond to anterior cues without crossing. Furthermore, when CoPA axons failed to cross the midline but grew toward the ventral spinal cord, they correctly turned dorsally and anteriorly as if they had crossed the midline (Figure 3B), indicating that they can respond to midline repellants as well as an anterior cue. Among the remaining CoPA axons that successfully crossed the midline, we observed a third phenotype. Occasionally, these axons turned anteriorly as they extended to the ventral midline as compared to wild-type CoPA axons, which do not turn before crossing (Figure 3C). These three phenotypes suggest that both anterior and dorsal pathfinding in CoPA is independent of midline crossing.

Since axon guidance toward the midline is a balance of attraction and repulsion, and *dcc* knockdown results in decreased attraction to the midline, we wanted to test whether increasing Robo-mediated repulsion would yield similar results. As a result, we assessed the role of *robo3* in CoPA pathfinding. Robo3.1 has been determined to allow midline crossing through inhibition of responsiveness to Slit-mediated repulsion [10,18]. Therefore, we hypothesized that in a *robo3* loss-of-function mutation we should observe increased midline repulsion and decreased crossing by CoPA axons. The *twt*^*tx209*^ mutant is a null allele for *robo3* that contains a nonsense mutation in the extracellular domain and should therefore abrogate the function of both C-terminal *robo3* splice isoforms (assuming that functionally redundant Robo3.1/3.2 forms exist in zebrafish [39]. *twt*^*tx209*^ heterozygous adults were incrossed, and progeny were analyzed for CoPA pathfinding defects. Interestingly, 54 ± 5.8% of progeny from a *twt*^*tx209*^ heterozygous incross displayed defective CoPA pathfinding (Figure 3D,E; *n* = 116). The occurrence of phenotypes in greater than 25% of *twt*^*tx209*^progeny suggested that abnormal pathfinding occurred in heterozygotes. To further investigate, *twt*^*tx209*^ adult heterozygotes were outcrossed to wild-type fish to generate a population of embryos in which 50% were heterozygous. In this population (*n* = 65), 27 ± 9.2% (Table 1) exhibited CoPA pathfinding defects, again indicating a non-recessive phenotype.

*twt*^*tx209*^ phenotypes fell into two categories. The first phenotype, affecting 16% of embryos from a *twt*^*tx209*^ heterozygote incross, consisted of an ipsilateral CoPA projection (Figure 3D). Since it was not seen in a heterozygote outcross, this presumably represents the homozygous null phenotype. In these embryos, CoPA axons grew ventrally toward the midline, but were unable to cross. Importantly, though axons failed to enter the midline, they were capable of pathfinding in the anterior and dorsal direction, ipsilateral to the CoPA cell body (Figure 3D). This phenotype was indistinguishable from the *dcc* morphant ipsilateral phenotype and is further evidence that midline crossing is not required for guidance in either the anterior or dorsal direction.

We observed a second defect that we termed “midline ascending.” In 41% of embryos from a *twt*^*tx209*^ heterozygote incross or 27% of embryos from an outcross, CoPA axons remained in the midline while ascending (Figure 3E). This phenotype presumably reflects a hypomorphic state of Robo3 function.

### DCC inhibits Robo2 activity prior to midline crossing

Through mutant and gene knockdown analysis, we determined that midline crossing is not required for dorsal and anterior pathfinding, which usually occurs after CoPA crosses the midline. To discern a molecular mechanism that could account for this, we investigated functional interactions between DCC and Robo2. The lack of ventral growth observed in *dcc* morphant embryos can be attributed to a deficiency in Netrin reception or an increase in responsiveness to repellant midline Slits. To test this latter possibility, we co-injected *robo2* translation blocking morpholinos with *dcc* morpholinos. When injected alone, we found that our *robo2* morpholino phenocopies the *robo2* (*ast*^ti272−/−^) null phenotype; 62.8 ± 2.9% (*n* = 177) of embryos injected with *robo2* MO exhibit the *ast*^ti272−/−^ phenotype (Table 1). Of these embryos, only 1.8 ± 1% (*n* = 177) exhibit axon guidance errors before crossing the midline (Table 1). As described above, 82.7 ± 4.6% (*n* = 157) of embryos injected with the *dcc* MO exhibit axon guidance defects before crossing the midline. Significantly, co-injection of *robo2* MO with the *dcc* MO reduces the percentage of embryos with the *dcc* pre-crossing phenotype on average by 13.7 ± 4.4% over three experiments (*p* = 0.036, *n* = 169). This partially epistatic relationship between *robo2* and *dcc* suggests that the failure of CoPA ventral growth in *dcc* morphants is at least partially due to Slit-mediated repulsion. Furthermore, these data suggest that ipsilaterally projecting commissural neurons in *dcc* knockdown embryos can respond to Slit signals through Robo2, which accounts for dorsal pathfinding in *dcc* morphants.

### Novel roles for Robo2 and Robo3 in establishment of commissure architecture

While studies on spinal commissural pathfinding have focused on the growth cone guidance in the dorsal-ventral axis, little is understood regarding mechanisms that underlie migration within the midline. In wild-type embryos, axons leave the midline in roughly the same anterior-posterior position in which they entered, creating a tight commissure in a precise location (Figure 2B). With CoPA axons, the commissure occurs directly ventral from the cell body, since CoPA ventral extension occurs at a 90-degree angle from the dorsal axis. The commissure can be defined as the area of the midline that CoPA axons occupy, which is devoid of the ventrally projecting ipsilateral axon or the dorsally projecting contralateral axon.

**Figure 4:**
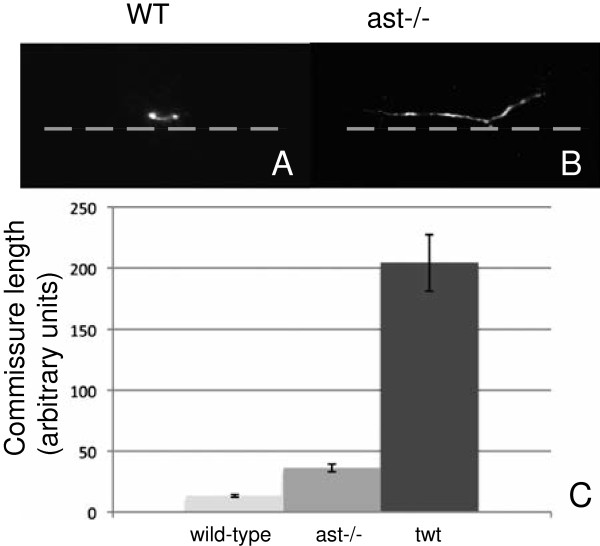
***robo2 *and *robo3 *are required for commissure formation. A**-**B** Confocal microscopic images of WT and *ast*^*−/−*^ commissures from individual CoPA axons (*lateral view*). *ast*^*ti272z*^ CoPA axons ascend in the midline while WT CoPA axons cross immediately. Anterior is to the *left*, dorsal is *up*. *Gray dotted line* indicates ventral midline of spinal cord. **C** Measurements of axon length within the midline of the spinal cord, in the anteroposterior axis (arbitrary units) in wild-type, *ast*^*ti272z*^, and *twt* embryos. *ast*^*ti272z*^ commissure length is 36 ± 3.2 (*n* = 7), which is 2.8 times that of wild type, which measures 13.3 ± 1.1 (*n* = 19) . In affected *twt*^*tx209*^embryos, the average length is roughly 15 times that of WT (204 ± 22.9; *n* = 9).

Due to our ability to analyze commissural neurons at the single-cell level, and the ability to simultaneously visualize both the dorsal-ventral and anterior-posterior axes, we were able to examine the functions of *robo2* and *robo3* in commissural architecture. Using confocal analysis, we determined that on average, *ast*^ti272−/−^ CoPA axons remained in the midline for 2.8 times the distance (*n* = 7) of wild-type axons (Figure 4A-C). Likewise, affected CoPA axons in *twt*^*tx209*^embryos remained in the midline 15 times the distance of wild-type axons (*n* = 9 for *twt*^*tx209*^, *n* = 19 for wild-type) (Figure 4C). In spite of extended growth in the midline, 100% of CoPA axons pathfind appropriately in the anterior direction (*n* = 16). Together these data indicate that Robo2 and Robo3 both contribute to define commissure position and width.

## Discussion

Commissures in the brain and spinal cord represent the physical scaffold upon which communication between the two sides of the nervous system relies. The vertebrate spinal cord is an ideal model to study commissural pathfinding, as its structure represents a more simplified version of the brain, yet the molecular mechanisms establishing the anatomy are similar. The zebrafish spinal cord provides the additional benefits of transparency, which allows axon pathfinding to be observed in many anatomical orientations. In spite of the anatomical simplicity of the spinal cord, the mechanisms that govern commissural pathfinding in this structure are complex. Commissural axon growth cones that are initially attracted to the midline must adjust their preference once crossed to allow exit from the ventral spinal cord and to establish subsequent pathfinding behaviors different from those just a few cell diameters away. It is known that specific guidance molecules regulate this process and that growth cones regulate their responsiveness based on previous experience [40-43]. Here, we have demonstrated that post-crossing behaviors in pioneer axons may represent a “default” state that must be actively inhibited pre-crossing, and that midline crossing is not obligately required for dorsal-ventral and anterior-posterior guidance.

With single-cell resolution, we have shown that commissural spinal pioneer neurons express axon guidance receptors in the DCC and Robo families. Using gene knockdown and mutant analysis, we have demonstrated that pioneer spinal commissural neurons utilize sequential guidance cues to navigate the midline. In mutant and gene knockdown conditions where midline crossing was prevented, pioneer neurons navigated the spinal cord as if midline crossing had been achieved, partially through Robo2 activity. This suggests that midline crossing per se is not required for reception of guidance cues normally received by contralateral growth cones. The mechanism that allows pre-crossing axons to normally ignore post-crossing cues relies in part on DCC-mediated inhibition of Robo2. Finally, we established novel roles for Robo genes in the establishment of commissure width.

### An *in vivo* demonstration of sequential utilization of guidance cues

Previous studies have demonstrated the requirement of multiple guidance systems in spinal cord commissural pathfinding. In higher vertebrates, Netrin/DCC signaling promotes ventral growth [28,44,45] and Rig-1/Robo3 (C-terminal splice isoform Robo 3.1) allows midline crossing by inhibiting responsiveness to Slit [10,18]. Post-crossing positioning of commissural neurons is dependent on Robo1, Robo2, and the Robo3 isoform Robo 3.2 [11,12,18]. However, these studies assayed behaviors on populations of commissural neurons, rather than at the single cell level. In addition, pioneer neuron behavior was not assessed.

**Figure 5:**
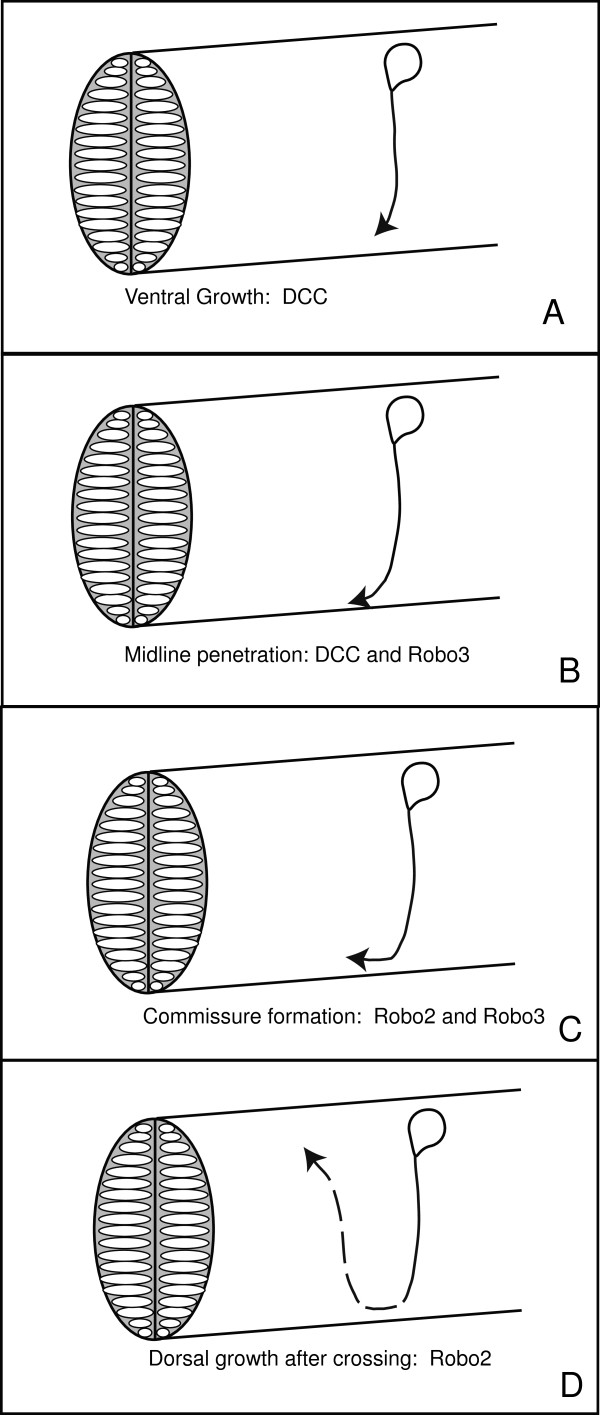
**Sequential roles for *dcc*, *robo3 *, and *robo2 *in CoPA pathfinding. A** CoPA utilizes DCC in order to navigate to the midline, through Netrin attraction, and inhibition of Robo2. **B** Once CoPA growth cones enter the ventral zone of the spinal cord, they require both Robo3 and DCC activity to penetrate the midline. **C** The formation of the ventral commissure in the spinal cord is determined through *robo2* and *robo3*. **D** Postcrossing dorsal growth is dependent on *robo2* activity.

Our data indicate that these molecular mechanisms are evolutionarily conserved in anamniote primary commissural neurons and are used sequentially at the single cell level. We have provided evidence that Robo and DCC family members are expressed in the pioneer commissural neuron, CoPA. Loss of function analysis demonstrates that CoPA utilizes DCC, Robo3, and Robo2 cues sequentially to make essential pathfinding decisions. In each case, we have identified multiple roles for each gene. We have observed that DCC is required for both ventral growth and midline penetration once growth cones have extended to the ventral spinal cord (Figures 5A-Bb. In addition, DCC inhibits Robo2 activity to prevent premature repulsion from the midline. Like DCC, Robo3 allows midline entry, but also promotes midline exit, as shown by a phenotype in which axons ascend in the midline (Figures 5B-C). Since *twt*^*tx209*^ is a null allele that removes all *robo3* isoforms, we predict that these distinct phenotypes may result from different Robo3 variants. For example, in amniotes, Robo3.1 is a midline gatekeeper through Robo1 inhibition, and Robo3.2 positions axons after crossing the midline [18]. The midline ascending phenotype may reflect loss of the zebrafish counterpart of Robo3.2, which could be responding to midline Slits similarly to Robo2. Interestingly, Robo2 also plays a role in facilitating midline escape, as *robo2* mutants exhibit longer retention of CoPA axons in the midline, though the phenotype is far less severe than in *robo3* mutants (Figure 5C-D). The redundancy of two genes in similar functions (effective midline expulsion) suggests the importance of precise placement and structure of the commissure, particularly as it pertains to segmentally reiterated structures in the nervous system.

In all loss-of-function conditions, though there were significant numbers of embryos that exhibited pathfinding errors, many CoPA axons exhibited normal trajectories. This is indicative of redundant guidance systems that ensure CoPA pathfinding. Candidates for these signals include other factors contributing to ventral extension, such as attraction through hedgehog family members [2], repulsion from roof plate derived BMPs [46], or attraction to Netrin through DSCAM [47-50]. Midline exit and post-crossing growth can be attributed to unknown roles of zebrafish Robo1 [11,12,51,52], which we were unable to test here because of the lack of a mutant allele or an effective morpholino, or to the commissural growth-promoting molecule stem cell factor (SCF) [53]. Anterior pathfinding could rely on non-canonical Wnt signaling, as has been demonstrated [54]. However, we were unable to observe CoPA pathfinding defects in either *wnt4* knockdown embryos or *wnt5b* (pipetail) mutants (our unpublished data).

### Midline crossing is not required for dorsal or anterior pathfinding

It is known that growth cone responsiveness changes with prior experience, affecting subsequent guidance decisions. For example, *in vitro* work on Xenopus spinal neurons has indicated that growth cones become desensitized (and resensitized) to Netrin, and this changing responsiveness is essential for navigation on Netrin gradients [42]. Also, chick dorsal root ganglion response to Laminin (increased growth versus growth cone stalling) is influenced by prior exposure to Laminin, or an electrical stimulus [41]. In explant studies, commissural neurons are sensitive to repellant Slits and Semaphorins only after crossing the midline [5]. While these studies clearly demonstrate the ability of growth cones to adapt *in vitro* and in explant cultures, our work suggests that prior exposure to guidance cues is not strictly required for subsequent signaling and pioneer axon pathfinding.

In *dcc* morphants, the lack of a ventral projection and midline crossing does not inhibit CoPA’s ability to grow anteriorly, which is the last major guidance decision of this axon. Therefore, CoPA growth cones likely do not rely on Netrin reception to respond to either an anterior attractant or posterior repellent. In fact, our data suggest that DCC function may normally inhibit anterior growth. Further, some CoPA axons in *dcc* morphants are able to reach the ventral spinal cord, at which point they make the appropriate dorsal-anterior turns as if they had crossed the midline. As post-crossing responsiveness to Slits and Semaphorins has been extensively demonstrated [5,10,11], we predict that the ability of *dcc* morphant axons to grow dorsally is due to an unidentified dorsal attractant, or premature responsiveness to Slit through Robo2. Again, this evidence suggests that prior growth cone experiences are not required for all subsequent decisions. This phenomenon is not specific to Netrin/DCC signaling as the same reasoning holds for *robo3* mutant axons, which extend dorsal-anteriorly in the absence of midline crossing. Consistent with our data, precerebellar neurons are capable of appropriate targeting in the absence of crossing which is caused by *robo3* downregulation [20]. Also, in belladonna (*bel*) mutants, retinotectal axons fail to cross the midline, but are capable of targeting the tectum albeit on the incorrect, ipsilateral side [55]. In *ast/bel* double mutants, pre-crossing pathfinding errors reveal a function for Robo2 prior to midline crossing [34] which is consistent with a pre-crossing mechanism that normally inhibits Robo2 function.

The contribution of segment maturation was not investigated in this study. However, it is possible that CoPA axons stall in various mutant and gene knockdown scenarios where midline crossing is prevented. In this model, the stalled CoPA axons would be unable to pathfind because of lack of appropriate post-crossing guidance cue availability (either through expression or receptor activation). Once these cues were available, CoPA would regain extension as if it had crossed the midline. As our analysis took place at 33 hpf (approximately 12 h after CoPA axonogenesis), we were unable to address this possibility. Time-lapse confocal analysis in live, labeled CoPA neurons would address this possibility.

### The inhibition of Robo2 by DCC is required for midline crossing

In both *robo3* mutant and *dcc* knockdown embryos, CoPA axons respond to guidance cues as if they have crossed the midline, suggesting that CoPA growth cones retain the ability to respond to Slit ligands as well as anterior cues, though this is normally masked to allow ventral extension. In amniotes, it has been shown that Robo3 inhibits Robo1, which accounts for increased Slit reception in pre-crossing axons [10]. However, a similar function has not been previously demonstrated with DCC. Here, we show that *dcc* and *robo2* double morphant embryos exhibit decreased ipsilateral pathfinding errors compared to *dcc* morphants, suggesting that DCC promotes ventral extension through inhibition of Robo2-mediated Slit repulsion. Since suppression of the ipsilateral phenotypes was incomplete (reduced by 13.7%), we predict that DCC has dual functions as both an attractive receptor for Netrin, as well as an inhibitor of Slit responsiveness, through Robo2.

### Commissure architecture relies on Robo2/Robo3

Our analysis has established roles of Robo genes in commissure architecture in the anterior-posterior axis. Wild-type CoPA axons cross the midline in a zone that is nearly perpendicular to the midline itself. In other words, cues present in the midline not only instruct commissural axons to cross, but establish tight commissure regulation which will lay the foundation for later arising commissural neurons. This mechanism supports adult spinal cord anatomy and topography within commissures. However, in *robo2* and *robo3* mutants, though pioneer commissures are formed, the anterior-posterior extent of the midline that CoPA axons occupy is significantly larger. This is likely due to defective Slit reception, which in addition to post-crossing repulsion, also mediates midline escape. While confined to the midline, *robo2* and *robo3* mutant axons correctly pathfind anteriorly and do not recross the midline.

Slit/Robo signaling has been previously implicated in the structure of commissures in the brain, though this likely occurs through a slightly different mechanism. For example, the optic chiasm is devoid of Slit, and the surrounding expression of Slit serves to restrict retinal growth cone navigation to a narrow zone [34,56-59]. In other brain commissures, for example the post-optic commissure in zebrafish and the corpus callosum in mice, axons also navigate the midline through avoidance of surrounding *slit* expression [60-63]. In contrast, Slit is expressed throughout the ventral spinal cord, including the ventral commissure, the site of commissural fiber crossing [6-8,64]. Unlike brain commissures, the ventral commissures in the spinal cord are segmentally repeated throughout the spinal cord. Additionally, spinal cord commissures are occupied by fewer axons. Thus, the timing of the switch in growth cone responsiveness is likely highly sensitive in the spinal cord, as any delay in processing guidance cues results in aberrant growth within the midline and wider commissures. In both *robo3* and *robo2* mutants, CoPA axons that remain in the midline too long eventually leave; however, they do not appear to correct their errors, as is observed in the optic chiasm [34]. While our analysis was limited to fixed specimens, it was performed 13 h after the pathway was established, suggesting that the opportunity for error correction has passed [21].

## Conclusions

In summary, these experiments have shown that pioneer commissural neurons possess an intrinsic ability to respond to a set of guidance cues that they normally only follow after midline crossing. The distinct pathfinding behaviors exhibited by commissural neurons, compared to their ipsilaterally projecting counterparts, must therefore derive primarily from molecular pathways functioning in the pre-crossing state. A unique system exists in these cells, temporarily blinding them to guidance cues that act more generally on the neuronal population.

## Methods

### Fish strains and mutants

Wild-type embryos were collected from natural matings of AB* or WIK. *robo2* mutants were generated by incrossing *ast*^ti272−/−^ adults [33]. *robo3* mutants were generated by incrossing *twitch twice* (*twt*^*tx209*^) carriers [65], identified by PCR genotyping as described by Burgess et al. [39].

*NBT:tau-GFP* embryos were obtained through incrossing homozygous or heterozygous *Tg(NBT:MAPT-GFP)*^*zc1*^ adults. This transgenic line was generated by injecting linearized NBT:tau-GFP plasmid [23] into one-cell wild-type embryos, raising, and crossing to screen for genomic integrations. In this plasmid, the *Xenopus Neuronal β-tubulin* promoter was placed upstream of a *tau (MAPT)-GFP* fusion construct, driving protein expression preferentially in axons of all neurons.

### Injection of *NBT:Tau-GFP*

50 pg of *NBT:Tau-GFP* plasmid DNA [23] was injected at the one-cell stage. At 24 h post-fertilization (hpf), GFP-positive CoPA neurons were identified based on morphological criteria including location of the cell body and axonal trajectory. Embryos with CoPA GFP fluorescence were fixed in 4% paraformaldehyde in 1× PBS overnight at room temperature.

### Morpholino injections

2 ng of *dcc* translation blocking morpholino, GAATATCTCCAGTGACGCAGCCCAT (start codon complement underlined [38] or 1 ng of *robo2* translation-blocking morpholino, TCCTGTCATAGTCCACATCCACACC), was injected at the one-cell stage using an ASI MPPI-3 (Applied Scientific Instrumentation) pressure injector. All morpholinos were obtained from Gene Tools, LLC.

### Double morpholino injections

*robo2* and *dcc* MOs were co-injected on the same day using the same needle for all injections to ensure reproducibility of dose. In one injection session, 2 ng of *robo2* MO was injected into one clutch of embryos, 3 ng of *dcc* MO was injected into a separate clutch of embryos, and a cocktail of 3 ng of *dcc* and 2 ng *robo2* MOs was injected into a third clutch of embryos. Phenotypes scored in double morphants were compared to single MO injections that were conducted on the same day with the same needle, which was rinsed when MOs were changed.

### *In situ* hybridization (ISH)

Probe synthesis and ISH were performed as described by [66]. Digoxigenin antisense RNA probes were visualized with BM Purple (Roche). We generated the following probes: *robo2* was generated by amplifying a 1,017-bp fragment from AB* cDNA using primers GTACAGGCAGATGTCAGG and GGAGTGGAGGATCTGTGT. *dcc* probe was generated as previously described [38]. The following probe was a gift: *robo3var2*[16].

### Immunofluorescence

The 3A10 antibody (DSHB) was used at a concentration of 1:25 on embryos at 33 hpf that had been fixed in 4% paraformaldehyde (in 1× PBS) for 3 h. Embryos were washed in PBTT (1× PBS with 0.5% Triton X-100 and 0.1% Tween-20). Cy3 conjugated AffiniPure Goat Anti-Mouse IgG (H + L) secondary antibody (catalog no. 115-165-003, Jackson ImmunoResearch Laboratories, Inc.) was added at a concentration of 1:200 overnight at 4°C. Embryos were washed 4× 15 min in PBT (1× PBS with 0.5% Triton X-100) in between antibody incubations.

### *Combined in situ* hybridization and immunofluorescence

Post-fixative washes were performed [3× 5 min in PTw (1× PBS + 0.1% Tween-20)], followed by 20 min incubation in 0.1% collagenase (Sigma-Aldrich C9891-100MG). Embryos were fixed in 4% paraformaldehyde for 20 min, followed by 4× 5-min washes in PTw. Embryos were incubated for 1 h at 65°C in prehybridization solution [50% formamide, 5× SSC, 0.1 mg/ml heparin (Sigma-Aldrich H3393-50 KU), 0.1 mg/ml torula yeast RNA (Sigma-Aldrich R6625-25 G), 0.1% Tween-20]. Prehybridization solution was removed and replaced with prehybridization solution plus either *robo2*, *robo3var2*, or *dcc* probe at concentrations of 1:100. Embryos were incubated overnight at 65°C in probe solution. The next day, embryos were washed 2× 30 min at 65°C in wash buffer 1 (50% formamide, 2× SSC, 0.1% Tween-20), followed by one wash (15 min) at 65°C in wash buffer 2 (2× SSC, 0.1% Tween-20). Final washes (2× 30 min at 65°C) in wash buffer 3 (0.2× SSC, 0.1% Tween-20) were performed, followed by 2× room temperature washes in PTw. Embryos were incubated in blocking solution [10% heat inactivated goat serum PBT (1× PBS and 0.5% Triton X-100)]. Upon block removal, embryos were incubated overnight at 4°C in 1:500 anti-GFP, rabbit IgG fraction (anti-GFP, IgG) 2 mg/ml, polyclonal (Invitrogen catalog no. A-11122), and 1:5,000 anti-digoxigenin-AP, Fab fragments (Roche Scientific, catalog no. 11093274910), diluted in block. Antibody solution was removed followed by washes (4× 15 min) at room temperature in PTw. After the last wash, embryos were incubated in room temperature BM Purple (Roche Scientific catalog no. 11442074001) overnight, in the dark, at room temperature. Embryos were washed 3× 5 min in PTw, followed by fixation for 20 min in 4% paraformaldehyde. Embryos were washed 3× 15 min in PBT. Cy3 Affinipure goat anti-rabbit IgG (H + L) secondary antibodies (Jackson ImmunoResearch catalog no. 111-165-003) were used at a concentration of 1:200 in 10% heat-inactivated goat serum-PBT. Embryos were incubated at 4°C overnight. Post-secondary Ab incubation was followed by 4× 15-min washes in PBT. Embryos were mounted as described above.

### Microscopy of embryos

After ISH or immunofluorescence, the yolks of embryos were removed through micro-dissection, and embryos were mounted on slides on their sides for lateral visualization of spinal cords. Embryos were mounted in SlowFade Gold antifade reagent (Invitrogen, catalog n. S36936). Embryos were visualized with either 40× or 60× Nomarski Optics for ISH, or 40× or 60× conventional fluorescence microscopy for analysis of wild-type, mutant, and morphant axon pathways.

### Confocal imaging

Confocal images of labeled neurons were obtained on either an Olympus FluoView 300 (Skidmore College) or Olympus FluoView 1000 (University of Utah). To obtain images of the left and right sides of the spinal cord, confocal projections were made by imaging from the location of CoPA cell bodies on one side of the spinal cord through to the CoPA cell bodies on the other side of the spinal cord. For images of one side of the spinal cord (to illustrate aberrant ipsilateral projections), confocal projections began at the CoPA cell body and terminated in the midline of the spinal cord. For images of the midline, projections were generated by imaging the midline of the embryo, as determined by the lack of CoPA cell bodies and lack of ventral projection, which extends from the CoPA cell body, on the lateral edge of the spinal cord.

### Quantification of *astray* phenotype

Each embryo was screened for the presence of post-crossing CoPA axons growing anteriorly in the ventral spinal cord parallel to the midline for one segment or longer. In order for an embryo to be scored defective for CoPA pathfinding, the phenotype must be present in more than one segment. Only CoPA neurons in segments adjacent to the yolk extension were scored to ensure consistency between embryos.

### Quantification of midline crossing in *astray* and *twt* mutants

Midline projection length was measured using either confocal projections of midlines (*ast*), or conventional fluorescent microscopic images (*twt*). Using ImageJ, we measured the length (along the anterior-posterior axis) of the spinal cord midline that was occupied by an individual CoPA axon.

### Statistics

For all experiments, at least three replicates were performed. Error bars and p-values were calculated based on SEM and two-tailed t-test, respectively.

## Abbreviations

CoPA: Commissural primary ascending; DLF: Dorsal longitudinal fasciculus; Hpf: Hours post fertilization; DCC: Deleted in colorectal cancer; ast: Astray; bel: Belladonna; NBT: Neuronal β-tubulin; twt: Twitch twice; MO: Morpholino; ISH: in situ hybridization.

## Competing interests

The authors declare that they have no competing interests.

## Authors’ contributions

JB wrote the manuscript, was involved in the conceptual design of experiments, carried out experiments and data analysis, imaged embryos, and performed statistical analysis. ML carried out *dcc* and *robo2* morpholino experiments. OBN performed expression studies in CoPA neurons; LK analyzed commissure width in astray and wild type; AC performed *dcc* morpholino experiments; BC performed *dcc* expression studies; PC analyzed *robo3* mutants; AL described the *dcc* morphant phenotype; CBC was involved in the conceptual design of experiments and editing of the manuscript; RD was involved in conceptual design of experiments, helped write and edit the manuscript. All authors read and approved the final manuscript.
